# Accuracy of Critical Shoulder Angle and Acromial Index for Predicting Supraspinatus Tendinopathy

**DOI:** 10.3390/diagnostics12020283

**Published:** 2022-01-22

**Authors:** Tzu-Herng Hsu, Che-Li Lin, Chin-Wen Wu, Yi-Wen Chen, Timporn Vitoonpong, Lien-Chieh Lin, Shih-Wei Huang

**Affiliations:** 1 Department of Physical Medicine and Rehabilitation, Shuang Ho Hospital, Taipei Medical University, New Taipei City 23561, Taiwan; 17324@s.tmu.edu.tw (T.-H.H.); 09442@s.tmu.edu.tw (C.-W.W.); 17304@s.tmu.edu.tw (Y.-W.C.); 17483@s.tmu.edu.tw (L.-C.L.); 2 Department of Orthopedic Surgery, Shuang Ho Hospital, Taipei Medical University, New Taipei City 23561, Taiwan; 11010@s.tmu.edu.tw; 3 Department of Orthopedics, School of Medicine, College of Medicine, Taipei Medical University, Taipei 11031, Taiwan; 4 Department of Physical Medicine and Rehabilitation, School of Medicine, College of Medicine, Taipei Medical University, Taipei 11031, Taiwan; 5 Rehabilitation Department, King Chulalongkorn Memorial Hospital, Bangkok 10330, Thailand; timpornvitoonpong@gmail.com

**Keywords:** shoulder, supraspinatus tendinopathy, critical shoulder angle, acromial index

## Abstract

Critical shoulder angle (CSA) is the angle between the superior and inferior bony margins of the glenoid and the most lateral border of the acromion. The acromial index (AI) is the distance from the glenoid plane to the acromial lateral border and is divided by the distance from the glenoid plane to the lateral aspect of the humeral head. Although both are used for predicting shoulder diseases, research on their accuracy in predicting supraspinatus tendinopathy in patients with shoulder pain is limited. Data were retrospectively collected from 308 patients with supraspinatus tendinopathy between January 2018 and December 2019. Simultaneously, we gathered the data of 300 patients with shoulder pain without supraspinatus tendinopathy, confirmed through ultrasound examination. Baseline demographic data, CSA, and AI were compared using the independent Student’s t test and Mann–Whitney U test. Categorical variables were analyzed using the chi-square test. A receiver operating characteristic curve (ROC) analysis was performed to investigate the accuracy of CSA and AI for predicting supraspinatus tendinopathy, and the optimal cut-off point was determined using the Youden index. No statistical differences were observed for age, sex, body mass index, evaluated side (dominant), diabetes mellitus, and hyperlipidemia between the groups. The supraspinatus tendinopathy group showed higher CSAs (*p* < 0.001) than did the non-supraspinatus tendinopathy group. For predicting supraspinatus tendinopathy, the area under the curve (AUC) of ROC curve of the CSA was 76.8%, revealing acceptable discrimination. The AUC of AI was 46.9%, revealing no discrimination. Moreover, when patients with shoulder pain had a CSA > 38.11°, the specificity and sensitivity of CSA in predicting supraspinatus tendinopathy were 71.0% and 71.8%, respectively. CSA could be considered an objective assessment tool to predict supraspinatus tendinopathy in patients with shoulder pain. AI revealed no discrimination in predicting supraspinatus tendinopathy in patients with shoulder pain.

## 1. Introduction

Supraspinatus (SS) tendinopathy is a type of tendon disorder characterized by pain and impaired function. It is related to degeneration, irritation, overuse, and poor strain mechanics [1,2]. Shoulder impingement syndrome is also believed to lead to SS tendinopathy [3]. Moreover, the causes of SS tendinopathy are variable and can be divided into intrinsic and extrinsic factors [3]. Intrinsic factors include age, excessive weight, and impaired biomechanics, including malalignments and decreased flexibility, causing degenerative changes and reduced strength of the tendon [4,5,6]. Extrinsic factors can be divided into primary and secondary impingement, which result from increased subacromial loading and muscle overload/imbalance, respectively [3,7,8,9,10]. Studies have reported that SS tendinopathy leads to poor sleep quality, low quality of life, and work absenteeism [11,12,13].

Rotator cuff tendinopathy is the most common cause of shoulder disorders [14], and its prevalence rates range from 5–10%, 30–35%, and up to 80% in people aged <20 years, 60–80 years, and >80 years, respectively [15,16,17]. Despite their variable etiology, supraspinatus tendinopathy is the most common among rotator cuff diseases, affecting 61.9% of men and 38.1% of women [18]. Hsiao et al. reported that subacromial impingement occurred at 7.77 per 1000 person-years in the military and observed that those aged >40 years had an increased risk of subacromial impingement, thereby leading to an increased risk of SS tendinopathy [19].

“Critical shoulder angle” (CSA), proposed by Moor et al., representing the inclination of the lateral extension of the acromion and glenoid on an anteroposterior (AP) radiograph [20], was reported higher in patients with degenerative rotator cuff tear than in those with non-rotator cuff tears [21]. Recent studies have also used CSA to predict supraspinatus tendon tear [22] and the risk of supraspinatus retear after surgery [23]. Furthermore, CSA along with age was found to predict cuff tear arthropathy, osteoarthritis, rotator cuff impingement, and calcified tendinitis [24]. On the other hand, “Acromial index” (AI), introduced by Nyffeler et al., representing the lateral extension of the acromion above the humeral head [25], has been revealed as a predictor of rotator cuff tear [21,26]. However, the ability of AI to predict the postoperative outcomes of rotator cuff tears is conflicting [27,28]. As Neer reported, 95% of rotator cuff tears might arise from SS tendinopathy, which is caused by the predisposition to the conditions of anatomic impingement [7].

Despite many studies evaluating the outcomes of rotator cuff disorders by using CSA and AI, no study has investigated the relationship between CSA and AI with SS tendinopathy. Therefore, this study aims to establish the association between CSA, AI, and supraspinatus tendinopathy, comparing the accuracy of CSA and AI in predicting supraspinatus tendinopathy.

## 2. Materials and Methods

### 2.1. Study Design and Participants

This study was designed as a retrospective case–control cross-sectional investigation and performed in a medical university hospital between January 2018 and December 2019. All participants were recruited from orthopedic and rehabilitation outpatient departments, and the Institutional Review Board of Taipei Medical University (N202011086) approved the study protocol. We applied the following inclusion criteria: (1) age between 20 and 80 years, (2) having shoulder pain, and (3) undergoing shoulder X-ray and ultrasound evaluation. The following exclusion criteria were applied: (1) previously underwent shoulder surgery around the shoulder; (2) having glenohumeral osteoarthritis and acromioclavicular arthritis, which could affect CSA and AI measurements; and (3) poor quality of shoulder radiographic images. Based on the findings of the shoulder ultrasound and physical examination (both painful arc and empty can test positive), participants were divided into the SS tendinopathy group and non-SS tendinopathy group. Baseline demographic data, such as age, sex, affected side, body mass index (BMI), history of diabetes mellitus, and hyperlipidemia, were obtained from medical charts.

### 2.2. Radiographic Evaluation for CSA and AI

**Figure 1:**
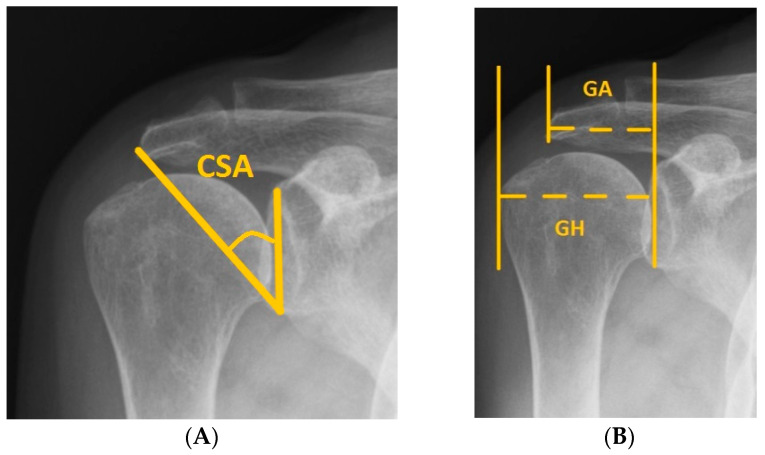
(**A**) The critical shoulder angle (CSA) is formed from a line connecting the inferior and superior borders of the glenoid fossa and another line connecting the inferior border of the glenoid with the inferolateral border of the acromion. (**B**) The acromial index is the ratio of the distance from the glenoid plane to the lateral border of the acromion (GA) to the distance from the glenoid plane to the most lateral aspect of the humeral head (GH). AI = GA / GH.

After demographic data collection, conventional AP shoulder radiographs were obtained on the day of the outpatient department visit. The image was taken with the patient in the upright standing position with a descending beam tilt of 20°. The shoulder AP image was obtained using a standardized protocol such that CSA could be measured accurately by clearly presenting the superior and inferior border of the glenoid fossa, and inferolateral border of the acromion. We adopted the CSA measurement protocol reported by Blonna et al. [29]. When the radiograph was not affected by rotation and overlapping of the anterior and posterior edges of the glenoid cavity, we defined it as having sufficient image quality for CSA assessment. Based on a previous study, the inter- and intra-observer reliability for measuring the CSA was excellent [30]. CSA was measured from the angle made by the superior and inferior bony margins of the glenoid and a line from the inferior bony margin of the glenoid to the most lateral border of the acromion (Figure 1A). As for AI, the GA was taken as the distance between the glenoid plane and lateral border of the acromion, and the GH was taken as the distance between the glenoid plane to the lateral aspect of the humeral head. AI was evaluated as the ratio of GA to GH (Figure 1B) [25].

### 2.3. Ultrasound Evaluation with Physical Examination of SS Tendinopathy

SS tendinopathy was confirmed through ultrasound and physical examination after the radiographic evaluation. The ultrasound and physical examination were performed by different physiatrists in our department. An experienced physiatrist, who was blinded to the result of the radiographic study of the shoulder, performed the evaluation for SS tendinopathy. Patients with SS tendinopathy displayed shoulder pain when performing shoulder abduction between 60° and 120°, and patients did not have radiation of pain to the neck or down the arm [31,32]. In addition, the empty can test was performed as the provocation test [33]. For ultrasound examination, patients assumed the modified Crass position with the palm on the iliac crest and the elbow directed posteriorly [34]. Sonography revealed thickening (>8 mm), hypoechogenicity, and heterogeneity in cases of SS tendinopathy [35]. According to a review article, ultrasound demonstrated a sensitivity of 79% and a specificity of 94% for the detection of rotator cuff tendinopathy [36].

### 2.4. Sample Size Estimation

G-Power 3.1 was used to estimate the sample size required for an analysis of two groups of independent means in the study. We input the effect size dz was 0.15, an alpha of 0.05, with a power of 0.95. We determined that a minimum total sample size of 483 was required to identify differences between the study groups. Considering the probability of patients’ data lacking and excluded due to the matching process, we enrolled more than 483 patients (653 patients) in our study to ensure adequate statistical power with an anticipated power of 0.95.

### 2.5. Statistical Analysis

Based on the ultrasound findings of SS tendinopathy, we divided all participants into the SS tendinopathy and non-SS tendinopathy groups. For reducing the influence of confounders, we match the demographic data such as age, sex, BMI, affected side, diabetes mellitus, and hyperlipidemia with a 1:1 ratio of both groups. The variables of age, sex, BMI, affected side, diabetes mellitus, hyperlipidemia, CSA, GA, GH, and AI are presented as the mean and number of patients. Continuous variables between the groups were compared using the independent Student’s t-test after the Kolmogorov–Smirnov test was performed to confirm these were normal distribution. If the data were not a normal distribution, we performed the Mann–Whitney U test to compare the mean value between the groups. The chi-square test was used for comparing categorical variables between the groups. We performed receiver operating characteristic (ROC) curve analyses of CSA and AI to estimate their accuracy for predicting SS tendinopathy. The cut-off points of optimal sensitivity and specificity of CSA and AI were determined by the Youden index. All statistical analyses were performed using Statistical Package for the Social Sciences (version 19.0; IBM, Armonk, NY, USA), and *p* < 0.05 was considered statistically significant.

## 3. Results

**Figure 2:**
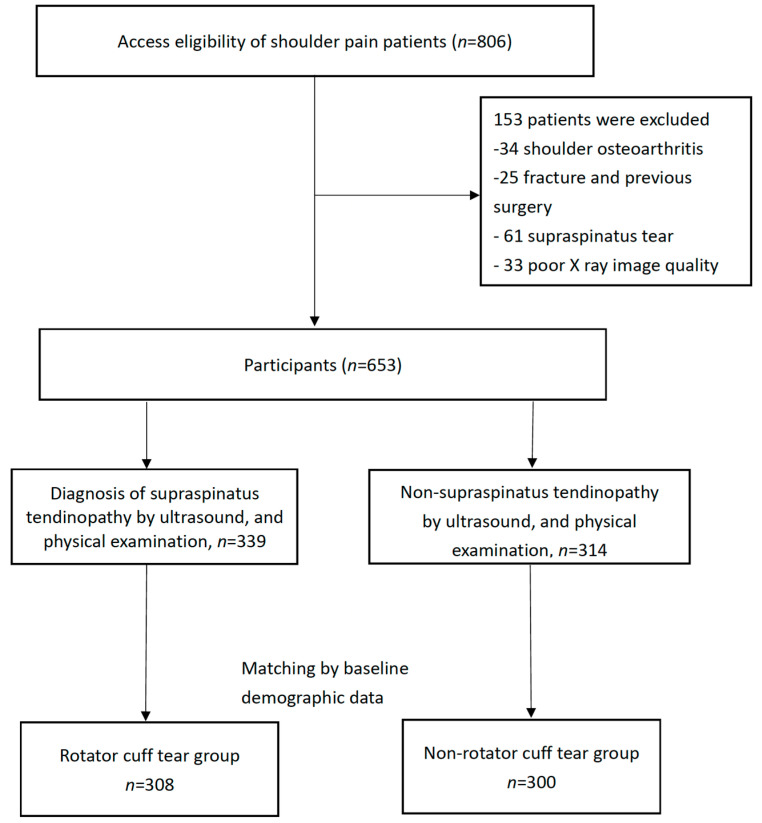
Flowchart of this study.

In total, 806 participants with shoulder pain met the inclusion criteria. Of them, 34, 25, 61, and 33 were excluded because of osteoarthritis, fracture, supraspinatus tear, and poor image quality for CSA measurement, respectively. Finally, 653 patients were included in this study. Based on the findings of ultrasound and physical examination, 339 patients were diagnosed as having SS tendinopathy; 314 participants having shoulder pain without SS tendinopathy comprised the non-SS tendinopathy group. For controlling the bias of the retrospective study, we matched the baseline variables between these two groups. Finally, 308 (148 men and 160 women) and 300 (143 men and 157 women) participants were included in the SS tendinopathy and non-SS tendinopathy groups, respectively (Figure 2).

No statistical differences were observed in demographic variables, such as age, sex, dominant side, BMI, diabetes mellitus, and hyperlipidemia, between these two groups (Table 1). Among these supraspinatus tendinopathy patients, there were 103 (33.4%) with supraspinatus calcific tendonitis, 90 (29.2%) with partial thickness tear, 89 (28.9) with supraspinatus tendinosis, and 26 (8.4%) with full thickness tear.

The results of the quantitative radiographic assessment presented in Table 2, demonstrated a significantly higher CSA in the SS tendinopathy group (40.29° ± 4.81°) than in the non-SS tendinopathy group (36.10° ± 3.55°; *p* < 0.001; 95% CI of difference: −4.9° to −3.5°). However, the GA, GH, and AI between the groups revealed no significant difference.

**Figure 3:**
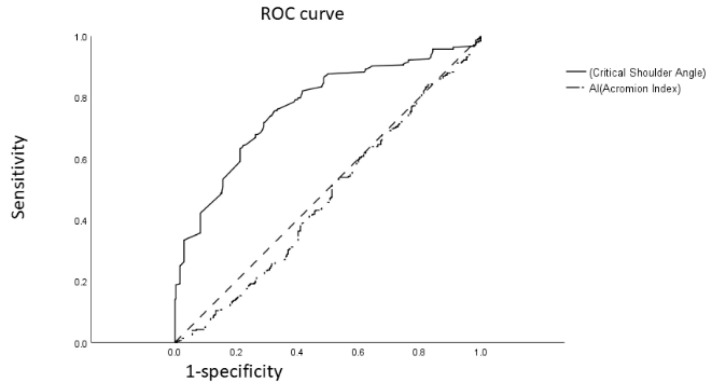
Receiver operating characteristic curve analysis of CSA degree and AI for predicting supraspinatus tendinopathy in patients with shoulder pain.

The ROC curve shown in Figure 3 with the area under the curve (AUC) for CSA was 76.8%, showing acceptable discrimination for patients with SS tendinopathy. However, the AUC of AI was 46.9% for predicting patients with non-SS tendinopathy, which showed no discrimination. According to the Youden index, the cut-off point of CSA was 38.11° with a sensitivity of 71.8% and a specificity of 71.0% in predicting SS tendinopathy. (Figure 3).

## 4. Discussion

To summarize, our results revealed that at a cut-off, CSA of 38.11°demonstrated acceptable discrimination for predicting SS tendinopathy in patients with shoulder pain. CSA showed a sensitivity of 71.8% and a specificity of 71.0%. However, AI revealed no discrimination for SS tendinopathy. This is the first study to investigate the diagnostic accuracy of SS tendinopathy in patients with shoulder pain by using CSA and AI on shoulder radiography.

Radiographic assessment is usually performed in clinics to evaluate patients with shoulder pain, with ultrasound possibly being required as a follow-up evaluation as determined by clinicians. Previous studies have revealed CSA as an objective assessment to predict rotator cuff tear, rotator cuff retear after surgery, shoulder impingement, calcified tendinitis, and glenohumeral osteoarthritis [23,24,37,38,39]. Our results first revealed CSA as a predictor of SS tendinopathy, which accounted for a proportion of patients with shoulder pain.

Immense stress is placed on the supraspinatus tendon, inserted under the acromion process, during shoulder abduction [7]. In addition, repetitive shoulder adduction places high loads on the supraspinatus tendon, thus causing SS tendinopathy [40,41]. Experimentally, increasing CSA would reduce the supero-inferior joint stability, leading to increased loads on the SS tendon to compensate for shoulder instability [40]. In addition, the workload of the rotator cuff increases in cases of high CSAs to counterbalance the ascending force of the deltoid, thus increasing mechanical burden and causing SS tendinopathy or tear [22]. SS tendinopathy can be a progressive disorder beginning with acute tendinitis, progressing to tendinosis with degeneration, and finally resulting in rotator cuff tear or rupture [7]. Numerous studies have demonstrated an association of CSA with rotator cuff tear or retear after surgery [23,25,39]. Theoretically, the mechanism detailed earlier could theoretically explain the relationship between CSA and SS tendinopathy.

Watanabe et al. and Heuberer et al. have reported a CSA of over 36.3° as a predictor of rotator cuff tear [24,42]. In addition, the more severe the rotator cuff tear is, the higher is CSA [43]. A recent systematic review by Zaid et al. demonstrated that several studies have reported significantly higher CSA in patients with rotator cuff tear compared to control groups [44]. Similarly, our results revealed significant differences in CSA between the SS tendinopathy and non-SS tendinopathy groups. This finding may be attributed to the same etiology that includes overload activity, muscle imbalance, shoulder impingement syndrome, and history of trauma [45,46]. In addition, SS tendinopathy is initially found before rotator cuff tear [7]; therefore, a high CSA could be a reasonable predictor of SS tendinopathy. Although SS tendinopathy may progress to supraspinatus tear, our study reported 38.11° as the cut-off of CSA for SS tendinopathy, which is higher than CSA in patients with rotator cuff tear (36° in earlier studies). This result may be attributed to the following reasons. First, our study included patients with shoulder pain, which increased the possibility of shoulder impingement caused by a high CSA; by contrast, previous studies were not limited to patients with shoulder pain [24,44]. Second, a study reported a higher CSA in degenerative rotator cuff tear than in traumatic rotator cuff tear (36.8° vs. 35.3°) [47]. Therefore, the difference in the proportion of the traumatic etiology of rotator cuff tear or SS tendinopathy may contribute to the difference in CSAs. Third, the different races may influence the type of build, which may cause different outcomes compared to previous studies.

In addition to CSA, a more lateral extension of the acromion is assumed to increase the force vector of the deltoid muscle, resulting in the subacromial abrasion of the rotator cuff tendon [25]. Based on this assumption, AI may be associated with rotator cuff tear or SS tendinopathy. Our results showed that AI is not suitable for predicting SS tendinopathy, although SS tendinopathy may potentially progress to rotator cuff tear. Miyazaki et al. reported that AI is associated with rotator cuff tear in Brazilians, but not in the Japanese population [48]; in addition, a different study revealed that AI may not be appropriate for predicting rotator cuff tear in the Taiwanese population [39]. Racial differences influencing unknown factors other than AI and impingement effect may be the reason for the conflicting results; thus, further investigation of such factors should be performed.

The strength of our study is using radiography to measure CSA for predicting SS tendinopathy in patients with shoulder pain, which was an objective assessment. CSA also demonstrated better accuracy than did AI in clinical applications for predicting SS tendinopathy. Nevertheless, our study has certain limitations. First, this was a retrospective study. To prevent heterogeneous data collection and bias of the radiographic image measurement, we controlled the demographic variables between the SS tendinopathy and non-SS tendinopathy groups by matching and standardizing the evaluation protocol of CSA and AI measurements. In addition, the assessor was blinded to the allocation of the group of patients with shoulder pain to reduce the evaluation bias. Second, morphologic parameters, such as low lateral acromion angles, anterior slope, and the shape of the acromion, were not analyzed in the study, which may affect rotator cuff pathologies [49,50,51]. Although these parameters of rotator cuff disease are debatable, the interaction among AI, CSA, and these parameters should be considered. Third, our study did not use MRI, which is considered a gold standard diagnostic tool, for detecting supraspinatus tendinopathy. However, considering cost, availability, safety, and efficiency of management, ultrasound is probably an option in most settings for the diagnosis of supraspinatus tendinopathy of daily practice. Finally, our study evaluated participants of a single race in Asia, and different races may affect results as previously mentioned. Finally, we evaluated only risk factors such as diabetes mellitus, hyperlipidemia, age, and BMI; factors such as biomechanical load in daily life and exercise should also be taken into account.

## 5. Conclusions

CSA could be used as an objective assessment tool to predict SS tendinopathy in patients with shoulder pain. Moreover, AI revealed no discrimination in predicting SS tendinopathy for patients with shoulder pain in our study. Although the AUC of CSA for predicting SS tendinopathy in patients with shoulder pain revealed acceptable discrimination, room for improvement remains. More extensive studies combined with other factors for predicting SS tendinopathy are required to strengthen the discrimination in the future.

## Figures and Tables

**Table 1:** Demographic and characteristics of Supraspinatus tendinopathy (SS tendinopathy) and Non-Supraspinatus tendinopathy (non-SS tendinopathy) groups.

Variables	SS Tendinopathy (*n* = 308)	Non-SS Tendinopathy (*n* = 300)	*p* Value
Age, y	57.1 ± 12.3	57.2 ± 13.0	0.870
Sex, n (male)	148	143	0.935
Evaluated side, n (dominant)	178	169	0.743
BMI, kg/m^2^	25.3 ± 3.5	25.2 ± 3.9	0.785
DM, n	59	65	0.481
Hyperlipidemia, n	28	32	0.587

Continuous data are shown as the mean ± standard deviation and categorical data as the number of patients; the *p* value was calculated using the Student’s t test for continuous variables and the chi-square test for categorical; variables; BMI, body mass index; DM, diabetes mellitus; VAS, visual analog scale.

**Table 2:** Quantitative radiographic assessment of Supraspinatus tendinopathy (SS tendinopathy) and Non-Supraspinatus tendinopathy (non-SS tendinopathy) groups.

X-ray Index	SS Tendinopathy (*n* = 308)	Non-SS Tendinopathy (*n* = 300)	*p* Value
CSA	40.29 ± 4.81	36.10 ± 3.55	<0.001 *
GA	3.76 ± 0.40	3.78 ± 0.38	0.377
GH	4.96 ± 0.54	4.94 ± 0.54	0.733
AI	0.76 ± 0.08	0.77 ± 0.08	0.088

Data were presented as the mean ± standard deviation; CSA, critical shoulder angle; GA, glenoid plane to the lateral border of the acromion distance; GH, glenoid plane to the most lateral aspect of the humeral head (GH); AI, acromial index * *p* < 0.05 by Mann–Whitney U test.

## Data Availability

Data available on request due to restrictions, e.g., privacy or ethical.
